# Trastuzumab-Conjugated pH-Sensitive Micelles Exhibit Antitumor Activity and Induce Mesenchymal-to-Epithelial Transition in Triple-Negative Breast Cancer Cell Lines

**DOI:** 10.3390/pharmaceutics17121554

**Published:** 2025-12-02

**Authors:** Crina Elena Tiron, Gabriel Luta, Razvan Ghiarasim, Adrian Tiron, Valentin Nastasa, Dragos Constantin Anita, Tore Geir Iversen, Tore Skotland, Kirsten Sandvig, Mihai Mares, Mihail-Gabriel Dimofte

**Affiliations:** 1 TRANSCEND Centre, Regional Institute of Oncology, 2-4 General Henri Mathias Berthelot Street, 700483 Iasi, Romania; transcendctiron@iroiasi.ro (C.E.T.); gabriel.luta@iroiasi.ro (G.L.); dimofte.gabriel@iroiasi.ro (M.-G.D.); 2 Centre of Advanced Research in Bionanoconjugates and Biopolymers, “Petru Poni” Institute of Macromolecular Chemistry, 41A Grigore Ghica Voda Alley, 700487 Iasi, Romania; ghiarasim.razvan@icmpp.ro; 3 Translational Research Center for Antimicrobial and Anticancer Agents, Iasi, Faculty of Veterinary Medicine, University of life Sciences, 8 Aleea Mihail Sadoveanu, 700489 Iasi, Romania; valentin.nastasa@iuls.ro (V.N.); dragos.anita@iuls.ro (D.C.A.); mihai.mares@iuls.ro (M.M.); 4 Department of Molecular Cell Biology, Institute for Cancer Research, Oslo University Hospital, 0379 Oslo, Norway; t.g.iversen@ous-research.no (T.G.I.); tore.skotland@ous-research.no (T.S.); kirsten.sandvig@ibv.uio.no (K.S.); 5 Centre for Cancer Cell Reprogramming, University of Oslo, 0379 Oslo, Norway; 6 Department of Biosciences, University of Oslo, 0316 Oslo, Norway; 7 Department of Surgery, “Grigore T Popa” University of Medicine and Pharmacy, 700115 Iasi, Romania

**Keywords:** TNBC, trastuzumab, drug delivery system, targeted delivery, EMT

## Abstract

**Background:** Despite improved knowledge on cancer prevention, progression, and treatment, the incidence of cancer is still increasing. Patients with highly aggressive triple-negative breast cancer benefit from chemotherapy as the only systemic therapeutic alternative. Here, we performed studies that demonstrate the effects of trastuzumab linked to nanostructures with pH-dependent release on triple-negative models. **Methods:** We assessed in vitro cell proliferation, migration, invasion, mammospheres, spheroids, and organoid formation of human and murine cell lines. Balb/c mice were used to investigate the in vivo anti-tumoral effects of functionalized nanostructures. Ex vivo samples and cell lines were used to investigate, using immunohistochemistry and Western blot, the modulation of key molecular pathways. **Results:** Using a human normal cell line and human and murine triple-negative breast cancer cell lines, we found that trastuzumab exhibits anti-tumoral properties on triple-negative breast cancer cell lines only when linked to pH-sensitive micelles. In addition, the data demonstrates that functionalized micelles induce mesenchymal-to-epithelial transitions, impairing the metastasis. **Conclusions:** Taken together, these results indicate that functionalization of micelles by linking trastuzumab may open the way of treating triple-negative patients with trastuzumab, a treatment which is currently in use for patients with Her2 overexpression. The functionalized micelles may be loaded with various molecules to further improve the anti-tumoral effects.

## 1. Introduction

Cancers are a major contributor to disease burden worldwide, and despite the recent advance in cancer research, forecast estimates that the global cancer burden will continue to grow for at least the next two decades [1,2]. The burden of cancer incidence and mortality is rapidly growing worldwide, with female breast cancer surpassing lung cancer as the most commonly diagnosed cancer [3]. New data supports the worldwide urgent need for comprehensive cancer prevention and to improve treatment to effectively reduce the global burden of breast cancer [4].

In breast cancer, patient prognosis and therapeutic decisions are conventionally based on clinicopathological variables such as tumor size, tumor grade, and nodal status, together with immunohistochemistry (IHC) biomarkers, such us estrogen receptor (ER), progesterone receptor (PR), and human epidermal growth factor receptor 2 (Her2) [5,6]. Her2 receptor is a member of a protein group known as epidermal growth factor receptors, which regulate cell growth and division. Normally, there is a low expression of Her2, as in Her2-negative tumors; Her2-positive (Her2+) tumors are defined by Her2 protein overexpression or gene amplification using IHC or fluorescence in situ hybridization, respectively [7].

A breast cancer subtype, Tripe-Negative Breast Cancer (TNBC), is a highly heterogeneous disease defined by the absence of ER, PR expression, and lack of Her2 overexpression [8]. Among breast cancers, TNBC exhibits a higher proliferation rate and higher incidence of metastases to the brain, liver, and lungs [9]. The lack of hormone receptors (ER and PR) and lack of Her2 overexpression prevent the use of usual targeted treatments, leaving chemotherapy as the only systemic therapeutic alternative [10]. Due to the fact that TNBC accounts for 15% to 20% of newly diagnosed breast cancer cases and its aggressive biological behavior and poor patient outcomes compared to hormone receptor-positive breast cancer, considerable effort has been made in recent years to classify TNBCs into different molecular subtypes according to genetic alterations and expression signatures and to uncover novel treatment targets, as reviewed recently [10,11].

The Her2+ subtype is frequently associated with invasion and metastasis [12]. Trastuzumab (Tzm) is the first clinically used humanized monoclonal antibody used for targeting overexpressed Her2 receptors. Although new molecules are currently used for targeted therapy in Her2+ patients [13], the toxicities of Her2 targeted therapies and the development of resistance to treatment challenge clinicians and researchers to search for novel and efficient treatment options by developing drugs with higher efficiency and by decreasing overall dose via targeted therapy.

In the search of new targeted therapies, many researchers are developing pH-sensitive nanocarriers with drug release at pH 4.5–6.5 [14,15,16]. In our investigations, we have developed pH-sensitive micelles with release at pH 7.2 [17]. We have focused our research on pH 7.2-sensitive nanostructures due to the fact that primary tumors (where pH could reach 6.5) are surgically removed, while metastases and the remaining tissue adjacent to primary tumors have a pH closer to the physiological one. Moreover, up to 40% of women develop local recurrence after surgery despite apparently tumor-free margins, and new data identified that morphologically normal breast may harbor early alterations that contribute to increased risk of cancer recurrence [18].

Recent data indicates that Tzm effects in Her2-negative breast cancer cell lines depend on Her2 phosphorylation [19]. Moreover, Tzm deruxtecan investigations resulted in significantly longer progression-free and overall survival in previously treated Her2-low breast cancer [20]. Tzm deruxtecan (formerly DS-8201), an antibody-drug conjugate consisting of a humanized anti-Her2 monoclonal antibody linked to a topoisomerase I inhibitor payload through a tetrapeptide-based cleavable linker (drug-to-antibody ratio, 8:1), can also effectively target tumor cells that express low levels of Her2 and can deliver its potent cytotoxic payload through the bystander effect to neighboring tumor cells heterogeneously expressing Her2 [21].

Aiming to restore sensitivity to Tzm in Tzm-resistant Her2+ cell lines, we have linked Tzm to the previously developed micelles in order to target cancer cells overexpressing Her2. Our initial in vitro investigations using SK-BR-3 cell line revealed that linking Tzm to micelles not only preserves therapeutic effects of Tzm but, in addition, seems to enhance this effect. Extending our investigations aiming to demonstrate if linking Tzm to micelles potentiates or not the therapeutic effects of Tzm, we have used both Her2+ cell lines (SK-BR-3, BT474, MDA-MB-361 and HCC1954) and TNBC cell lines as negative controls (human MDA-MB-231 and murine 4T1). As expected, free TZM did not reduce cell viability of the tested TNBC cell lines, but, to our surprise, Tzm linked to micelles significantly impaired cell viability of the TNBC cell lines.

In the current manuscript, we have investigated the effects of Tzm linked to micelles (Tzm-PEG-PHis) on TNBC cell lines by in vitro 2D and 3D models (mammospheres assay, monoculture and coculture Matrigel assay) and in vivo Balb/c-4T1 animal model. The in vitro biological effects we have seen in MDA-MB-231 and 4T1 cell lines are not observed in the widely used normal human breast cell line MCF-10A. Details regarding the preparation and characterization of Tzm-PEG-PHis are found [22].

## 2. Materials and Methods

### 2.1. Functionalized Nanostructures Development

The PEG–PHis copolymers used for micelle assembly were thoroughly characterized by HPLC, MS, and MALDI–MS, as detailed in our previous study [22].

#### 2.1.1. Functionalization of H_2_N-PEG2K-PHis32-COOH with Sulfo-SMCC

H_2_N–PEG2K–PHis32–COOH (20 mg) and sulfo-SMCC (6.70 mg, molar ratio 1:5) were dissolved in 2.6 mL of PBS (pH 7.4). The solution was sonicated for 1.5 h while maintaining the pH between 7 and 8, followed by gentle stirring for 24 h at 25 °C. The reaction mixture was purified using Amicon Ultra-4 centrifugal filter units (3 kDa MWCO) by three consecutive washes with deionized water (15 mL each) to remove unreacted components. The obtained product, Linker–PEG2K–PHis32–COOH, was lyophilized to a white powder and stored at −20 °C until use.

#### 2.1.2. Functionalization of H_3_CO-PEG2K-PHis32-NH_2_ with RBITC

To obtain fluorescently labeled copolymer, H_3_CO–PEG2K–PHis32–NH_2_ (20 mg) was dissolved in 1.6 mL of PBS (pH 7.6) and mixed with RBITC (4 mg, dissolved in 1 mL DMSO; molar ratio 1:2:5). The pH was adjusted to 8 with 0.1 M NaOH, and the mixture was stirred for 24 h at 25 °C in the dark. The reaction mixture was purified by diafiltration (Amicon Ultra-4, 3 kDa MWCO) using three washing cycles with deionized water (15 mL each). The purified copolymer, H_3_CO–PEG2K–PHis32–RB, was lyophilized to a pink powder and stored at −20 °C.

#### 2.1.3. Thiolation of Trastuzumab (TZM)

Native trastuzumab (0.5 mg) was dissolved in 500 µL of 0.1 M sodium phosphate buffer containing 5 mM EDTA (pH 8.0). A fresh solution of Traut’s reagent (2-iminothiolane hydrochloride, 2 mg/mL in deionized water) was prepared, and 23.2 µL of this solution (46.4 µg) was added to the antibody. The mixture was gently shaken for 4 h at 25 °C and purified by filtration (Amicon Ultra-4, 10 kDa MWCO) with three washes using 1 × PBS (1.5 mL each). The resulting thiolated antibody (TZM–SH) was adjusted to 1 mg/mL in PBS containing 5 mM EDTA (pH 7.6) and stored at 4 °C until further use.

#### 2.1.4. Preparation of PEG-PHis Micelles

PEG–PHis micelles were prepared as follows: H2N–PEG2K–PHis32–COOH (30 mg) and Linker–PEG2K–PHis32–COOH (10 mg) were dissolved in 200 mL of phosphate buffer (10 mM, pH 7.6, 0.5 mM EDTA), sonicated for 1 h, and stirred for 24 h at 25 °C to obtain non-functionalized micelles (L–PEG2K–PHis32). For the preparation of rhodamine B-labeled micelles (L–PEG2K–PHis32–RB), 30 mg of H3CO–PEG2K–PHis32–RB and 10 mg of Linker–PEG2K–PHis32–COOH were dissolved in 100 mL of the same buffer and processed under identical conditions, protected from light. To conjugate trastuzumab (TZM), 800 µg of TZM–SH (1 mg/mL) were added to 100 mL of each micellar dispersion to achieve a final antibody concentration of 4 µg/mL, followed by 24 h of gentle stirring at 25 °C to enable covalent coupling, yielding TZM–PEG2K–PHis32 and TZM–PEG2K–PHis32–RB micelles.

#### 2.1.5. Scanning Transmission Electron Microscopy (STEM)of Micelles

Morphological analysis was performed using a Verios G4 UC (Thermo Fisher Scientific, Waltham, MA, USA) in STEM mode at 30.00 kV with a STEM 3+ detector (bright field mode). Samples were deposited from an aqueous solution at 200 µg copolymer per mL in 1X PBS pH = 7.6 with 5 mM EDTA onto 300 mesh Lacey carbon-coated copper grids. Subsequently, solvent removal was performed under vacuum conditions, and micelle diameters, for both unfunctionalized and functionalized micelles, were measured using ImageJ 1.52i software.

### 2.2. Two-Dimensional Cell Cultures

MDA-MB-231 (triple-negative human breast cancer, ATCC), 4T1 (triple-negative murine mammary cancer, ATCC), and MCF-10A (human breast epithelial, ATCC) were cultured as described by Tiron et al. [23].

#### 2.2.1. Cell Viability and Mitochondrial Activity

Cell proliferation: Cells were plated on 96-well plates in eight replicates/treatment (3000 cells per well). After 72 h of incubation, allowing the pH to reach 7.2, the cells were treated as described in the figure legends. After another 72 h of incubation, CellTiter-Blue^®^ Reagent was added (50 μL of CellTiter-Blue for 100 μL of media). Viability rate was determined by absorbance using a multiplate microplate reader (FilterMax F5, Molecular Device, Sunnyvale, CA, USA).

For the mitochondrial assay, cells were incubated as before for 72 h with 2 µg/mL Tzm-PEG-PHis or free Tzm and then subjected to 1 μg/mL MitoTracker Red (Molecular Probe, Eugene, OR, USA) and incubated for 30 min before the fluorescence of the resultant solutions was determined at 590 nm using a multiplate microplate reader.

#### 2.2.2. Migration and Invasion Assay

Cytoselect 24-well cell migration and invasion assay (Cell Biolabs, Inc. CBA-100-C, San Diego, CA, USA) were used to perform these investigations, according to the manufacturer’s instructions. In total, 5 × 10^5^ cells per well were seeded in a serum-free medium supplemented with 0.1% BSA. Treated groups contained 2 µg/mL Tzm-PEG-Phis in the inserted medium. Non-migrated cells were removed with a cotton swab at twenty-four hrs after seeding, while non-invading cells were removed at forty-eight hrs from seeding. The remaining cells were fixed, stained with DAPI, and analyzed by fluorescence microscopy (Zeiss Axio Observer Z.1 microscope, TissueGnostics rig, Vienna, Austria). Sixteen images were acquired per well at 10× magnification using TissueFAXS 6.146 software (Vienna, Austria) and quantified by using ImageJ 1.52i software (National Institutes of Health, Wayne Rasband, Bethesda, MD, USA).

#### 2.2.3. EdU Assay

The Click-iT EdU Alexa Fluor 647 Imaging Kit (C10340, Thermo Fisher Scientific, Waltham, MA, USA) was used according to the manufacturer’s instructions. EdU (5-ethynyl-2′-deoxyuridine) provided in the kit is a nucleoside analog of thymidine and is incorporated into DNA during active DNA synthesis. At 72 h from concomitant administering of EdU and treatment, fluorescent dye was protected by using ProLong Gold Antifade Mountant with DAPI (Thermo Fisher Scientific P36941, Waltham, MA, USA). Using the Zeiss Axio Observer Z1 Microscope from TissueGnostic rig and TissueFAXS 6.146 software (Vienna, Austria), we acquired pictures at 20× magnification. Quantitative fluorescence cell analysis was performed using TissueQuest 6.0.1.126 software (Vienna, Austria).

### 2.3. Three-Dimensional Cell Culture Investigations

#### 2.3.1. Three-Dimensional Mammospheres Assays

Mammosphere cultures of MCF10A, MDA-MB-231, and 4T1 cell lines were performed as previously described by Dontu et al. [24]. Single cells were plated in ultra-low attachment plates (Corning, Acton, MA, USA) at 20,000 viable cells/mL in defined medium. After 72 h of incubation, the cells were treated with 2 or 4 μg/mL free Tzm/Tzm-PEG-Phis. After 14 days, mammospheres were fixed with 4% paraformaldehyde, stained with 4,6-diamidino-2-phenylindole (DAPI—nuclear stain) at 4 °C, and imaged using a fluorescence microscopy (Zeiss Axio Observer Z.1 microscope, TissueGnostics rig, TissueFAXS 6.146 software) (Vienna, Austria). Average area of mammospheres per well (8 wells per group) was quantified using ImageJ 1.52i software (National Institutes of Health, Wayne Rasband, Bethesda, MD, USA). Graphpad Prism 6.0 (GraphPad, La Jolla, CA, USA) was used for statistical analysis, and the following symbols are shown to report established statistical significance: **** *p* < 0.0001.

#### 2.3.2. Three-Dimensional Matrigel Assays

The 3D monoculture Matrigel assays were conducted with 1000 cells seeded in Ibidi plates, between 2 layers of Matrigel (Matrigel Matrix, Growth Factor Reduced—BD Biosciences, Franklin Lakes, NJ, USA). After 72 h of seeding, 3D embedded cells were treated with 2 μg/mL Tzm-PEG-PHis and cultured 21 days before microscopy analysis (Zeiss AxioObserver Z1 microscope Zeiss, Oberkochen, Germany; TissueGnostic rig, TissueFAXS 6.146 software, Vienna, Austria). Three weekly treatments (2 µg/mL Tzm-PEG-PHis) have been administered. For 3D Matrigel coculture assay, tumoral cells have been cultured together with fibroblasts and human umbilical vein endothelial cells (HUVEC) cells in a ratio of 1:1.5:1.5.

In a second experimental design, parental TNBC cell lines and Axl knockdown TNBC cells (shAxl2), a generous gift from James Lorens (BerGen Bio AS, Bergen, Norway), were cultured in the same manner but received one single 4 µg/mL Tzm-PEG-PHis treatment, and the experiment ended 14 days after treatment administering.

### 2.4. Tumor Model

The experiments were approved by the Ethical Committee of the Faculty of Veterinary Medicine, University of Life Sciences “Ion Ionescu de la Brad”, Iasi, and were performed in accordance with the European legislation on the protection of animals used for scientific purpose and with authorization from the National Sanitary Veterinary and Food Safety Authority (no. 394/23.03.2023). Female BALB/c mice (8–10 weeks old; Cantacuzino Institute, Bucharest, Romania) were used. The mice were housed in the animal facility at the Faculty of Veterinary Medicine, University of Life Sciences “Ion Ionescu de la Brad”, Iasi, in individually ventilated cages (IVCs) in a climate-controlled: 20 ± 4 °C, 50 ± 5% relative humidity and 12 h light/dark cycles, containing shaving bedding material, with regular rodent chow and water ad libitum.

4T1 mouse breast carcinoma cells were suspended in MEM-EBSS medium/Matrigel (1:1) (1 × 10^6^ in 50 μL) and injected into the mammary fat pad of female BALB/c mice under deep anesthesia (with 2% isoflurane). Tumor growth was monitored two times/week. At 2 weeks after tumor cells inoculation, mice begin treatment protocol via intraperitoneal injection. Mice were euthanized 5 weeks after tumor cell inoculation by neck dislocation under deep anesthesia (with 5% isoflurane). For fluorescent biodistribution investigation, we used the IVIS Spectrum in vivo imaging system (PerkinElmer), which employed a back-thinned charge-coupled device, cooled to −90 °C to achieve maximum sensitivity. Data analysis of IVIS spectrum data was performed using Living Image software version 4.7.3. To support absolute quantitation, the system measured dark charge during down-time and ran a self-calibration during initialization. The primary tumors and different organs were retrieved from the mice and preserved in 10% paraformaldehyde (Sigma-Aldrich) for further analysis (histopathology and immunohistochemistry).

### 2.5. Immunohistochemistry (IHC)

IHC was performed on 4 µm-thick sections of formalin-fixed, paraffin-embedded tissues. Sections were deparaffinized and rehydrated. Epitope recovery was performed by heating until reaching boiling point at 360 W and then maintained up to 20 min at 180 W in target retrieval solution buffer (pH 6) using a microwave oven. Endogenous peroxidase activity was blocked using peroxidase blocking solution (Dako, S202386-2, Santa Clara, CA, USA) for 15 min in the dark, at room temperature. The slides were incubated over night at 4o C with primary antibodies diluted in antibody diluent (Dako, K800621-2, Santa Clara, CA, USA): anti-Akt3 (Rockland, 200-301-E75, 1:250 Philadelphia, PA, USA), anti-phospho-p44/42 (Cell Signaling, D13.14.4E,1:200 Danvers, MA, USA), anti-vimentin (Cell Signaling, D21H3, 1:200 Danvers, MA, USA), and anti-Axl (1H12, 1:200, a generous gift from James Lorens, BerGenBio, Bergen, Norway). The incubations of the rabbit secondary antibody (Dako, K800921-2 Santa Clara, CA, USA) and of the streptavidin–horseradish peroxidase from Dako were performed at room temperature for 30 min each. After incubation with 3,3’-diaminobenzidine (Dako), slides were counter-stained with Hematoxylin (ThermoScientific, 7211, Waltham, MA, USA). TissueFAXS 6.146 software (Vienna, Austria) was used to acquire serial pictures and rebuild stained sections in digital format. Analysis for investigated markers expression was performed using HistoQuest 6.0.1.127 software (Vienna, Austria). IHC markers were investigated at the invasion front of primary tumors and in lungs metastases.

### 2.6. Western Blot

The Bradford method was used to measure protein levels in homogenate samples. Equal amounts of protein (35 µg/well) were loaded onto SDS-PAGE gels (10%) and ran at constant 100 V. At a constant voltage of 100 V for 1 h, separated proteins were transferred to a nitrocellulose membrane (Sigma-Aldrich Whatman Protran nitrocellulose transfer membrane, St. Luis, MO, USA). The resulting membranes were blocked on a gyro-rocker for 1 h at room temperature using a blocking buffer (BB) containing TBST (Tris-buffered saline/0.1% Tween 20) and 5% BSA or 5% non-fat dry milk. The primary antibodies were dissolved in BB containing 3% BSA: anti-Akt3 (Rockland, 200-301-E75, 1:1000 Philadelphia, PA, USA), anti-phospho-p44/42 (Cell Signaling, D13.14.4E,1:500 Danvers, MA, USA), anti-vimentin (Cell Signaling, D21H3, 1:2000 Danvers, MA, USA) and anti-Axl (1H12, 1:500), anti-E-cadherin (Santa Cruz Biotechnology, G-10, sc-8426, 1:500 Dallas, TX, USA), anti- β-catenin (Santa Cruz Biotechnology, E-5, sc-7963, 1:500 Dallas, TX, USA), and anti- β-actin (Cell Signaling, 8H10D10, 1:2000 Danvers, MA, USA). The blots were incubated with constant shaking at 4 °C overnight. After the membranes were washed three times with TBST, we incubated them for 1 h at room temperature in horseradish peroxidase-conjugated secondary antibody dissolved in TBST (dilution 1:5000) and washed them again three times with TBST. Proteins were visualized using WesternSure PREMIUM Chemiluminescent Substrate (Li-Cor, Lincoln, NE, USA) and Image Studio Digits software provided by Li-Cor (Lincoln, NE, USA).

### 2.7. Statistics

GraphPad Prism 6 (GraphPad, La Jolla, CA, USA) software was used for statistical analysis. Grouped analyses were performed using one-way ANOVA test for other statistical analysis. Quantitative data for statistical analysis were expressed as mean ± SEM (shown as error bar). Significance was established for *p* < 0.05.

## 3. Results

### 3.1. Synthesis and Morphological Analysis of Micelles

**Figure 1:**
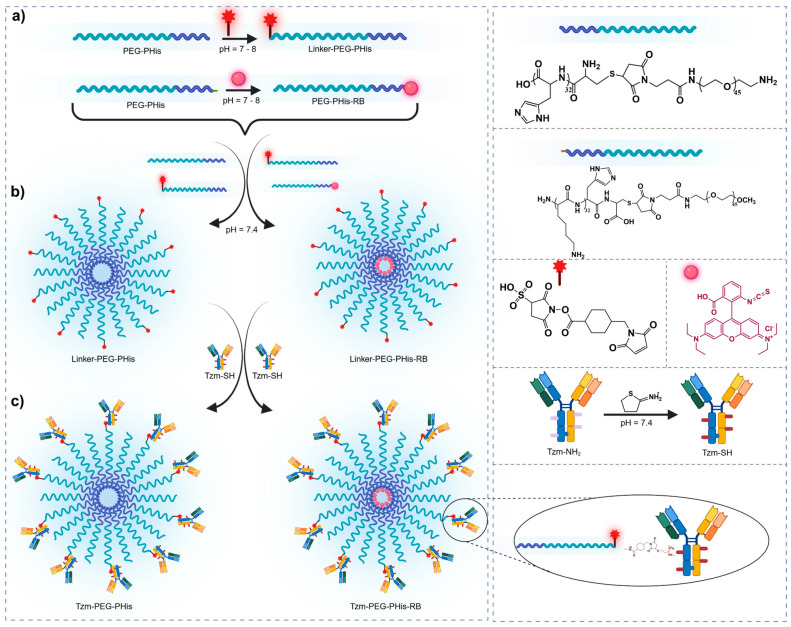
Schematic representation of the functionalized micelles development. (**a**) Maleimide functionalization of PEG termini and RB conjugation to the PHis terminal Lys, (**b**) Self-assembly of functionalized copolymers into micelles: unloaded (PEG-PHis) and RB-labeled (PEG-PHis-RB) and (**c**) TZM functionalization of micelles: TZM-PEG-PHis and TZM-PEG-PHis-RB.

The pH-sensitive micelles were synthesized employing an adapted method, as previously reported by our team [17]. Specifically, we utilized poly(L-histidine)-poly(ethylene glycol) block copolymer sequences (PEG-PHis) containing a polyethylene unit of 2 kDa and a poly(L-histidine) unit consisting of 32 repeating monomers. Subsequently, these micelles were further covalently functionalized with variable amounts of Tzm to yield Tzm-functionalized micelles (Tzm-PEG-PHis). The development of the functionalized micelles is graphically represented in Figure 1.

The morphology of the obtained micelles was elucidated using scanning transmission electron microscopy (STEM) (Figure S1). In general, the sizes obtained by microscopy are smaller than those obtained by DLS. This size reduction can be attributed to the dehydration of the micelles that occurs during the sample preparation process for STEM analysis. For the PEG-PHis sample (Figure S1a), homogeneous spherical micelles with an average diameter of 59.49 ± 9.71 nm were observed. The covalent binding of the linker to the surface of the micelles (Linker-PEG-PHis, Figure S1b) led to an increase in diameter to 78.56 nm ± 7.42 nm while maintaining their characteristic spherical shape. Later, the application of a layer of Tzm molecules on the surface of the micelles (Tzm-PEG-PHis) preserved their spherical shape (Figure S1c), resulting in an average diameter of 104.05 nm ± 11.98 nm. In the case of this sample, analyzing the STEM images (c) and (d) in Figure S1d, one can observe the attached Tzm molecules on the surface of the micelles, which again confirms the covalent attachment of Tzm to the surface of the micelles.

### 3.2. Anti-Cancer Investigations on Mono Layer (2D) In Vitro Cell Cultures

#### 3.2.1. Cell Viability, Mitochondrial Activity and Cell Proliferation

In clinical practice, Tzm is administered for Her2+ patients in a dose of 8 mg/kg (initial dose) in monotherapy, with subsequent decrease in dosage during Herceptin treatment [25]. In one experimental setup using Her2+ positive breast cancer cell lines, triple-negative breast cancer MDA-MB-231 cell line was used as negative control for investigation of Tzm therapeutic effects. As expected, Tzm alone did not present any impact on cell viability of MDA-MB-231 cancer cells. However, Tzm linked to micelles (Tzm-PEG-PHis) significantly impaired cell viability at 72 h from treatment administering. In order to validate the impact of Tzm-PEG-PHis in the MDA-MB-231 cell line, we tested various concentrations of free Tzm and the corresponding Tzm-PEG-PHis concentrations (Figure S2). Tested concentrations do not significantly impair cell viability of the normal breast cell line MCF-10A (Figure S2A). Again, empty micelles (PEG-PHis) and free Tzm do not decrease cell viability of MDA-MB-231 cells. To our surprise, concentrations of 2, 1 and 0.5 µg/mL Tzm-PEG-PHis had similar efficiency to decrease cell viability (Figure S2B), while at 0.25 µg/mL Tzm-PEG-Phis, there was no effect.

**Figure 2:**
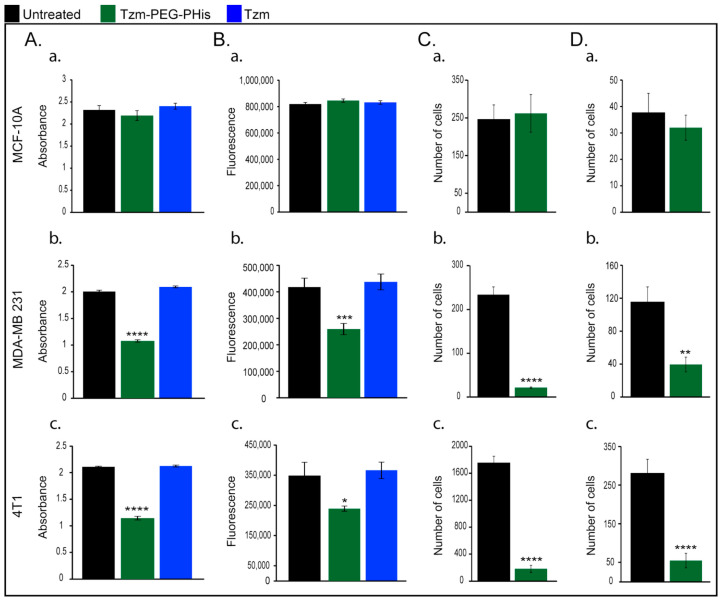
In vitro investigations. (**A**). cell viability; (**B**). mitochondrial activity; (**C**). Migration; (**D**). invasion; (**a**). MCF-10A; (**b**). MDA-MB 231; and (**c**). 4T1. * *p* = 0.0400; ** *p* = 0.019 *** *p* = 0.0004; **** *p* < 0.0001.

Next, we repeated the investigations on cell viability using 2 µg/mL Tzm-PEG-PHis (2 mg/kg) or 2 µg/mL free Tzm, but we added, for these investigations, the 4T1 cell line murine TNBC cell line (Figure 2). Although, in human TNBC cell line, the impact on cell viability occurred even at lower concentrations, we chose to focus on the 2 µg/mL concentrations on the next experiments because we cannot predict the interaction of human-designed Tzm with murine cells and the treatment efficiency in 3D assays. At 72 h from the start of the treatment, the new experiment endorsed our previous results on normal MCF-10A and MDA-MB-231 cell line (Figure 2A(a,b)) and revealed a significant impairment of 4T1 cell viability only in the case of Tzm-PEG-PHis (Figure 2A(c)).

Recent papers reviewed the involvement of mitochondrial metabolism in tumor growth, based on both human and mouse studies [26,27]. Taking into consideration the fact that mitochondria play a central and multi-functional role in malignant tumor progression, we next investigated the mitochondrial activity upon treatment with 2 µg/mL Tzm-PEG-PHis or free Tzm treatment (Figure 2B). In line with cell viability data, mitochondrial activity is not impaired in normal MCF-10 A (Figure 2B(a)), while in TNBC cell lines, it is significantly reduced only in the Tzm-PEG-PHis treated groups (Figure 2B(b,c)).

Cell viability assay reflects a cell population, which in turn represents a balance of cell proliferation and cell death. Using EdU assay, a novel alternative to the classical BrdU assay, we labeled the cells that undergo cell proliferation during the 2 µg/mL Tzm-PEG-PHis treatment administration (Figure S3). The cells that did not proliferate are negative for EdU (blue staining of nuclei), while positive EdU cells (red fluorescence staining) are cells resulting from cell division after EdU was added into cell media. Pooling together the number of total cells and the corresponding number of EdU-positive cells from several pictures acquired for each group, we find that 76% are EdU-positive in the case of untreated normal MCF-10A cells (Figure S3A,B(1)), 69% are EdU-positive in 2 µg/mL Tzm-PEG-PHis-treated MCF-10A group (Figure S3A,B(2)), approx. 22% in the case of 2 µg/mL Tzm-PEG-PHis-treated MDA-MB-231 (Figure S3A,B(3)), and 37% in the case of 2 µg/mL Tzm-PEG-PHis-treated 4T1 cells (Figure S3A,B(4)). The data shown demonstrates that 2 µg/mL Tzm-PEG-PHis reduce cell proliferation. In addition, fluorescence data shows reduced size of nuclei in TNBC treated cells.

In one of the few papers that investigates Tzm effects on Her2-negative cancer cell lines [19], the authors show that, in the case of TNBC cell lines, the effect of Tzm depends on Her2 phosphorylation. In TNBC cell lines, MDA-MB-468 and BT-549, which have phosphorylated Her2 (Y877), Tzm decreases proliferation but at doses much higher than the ones used in our experiments, and with lower efficacy as compared to Her2+ cell lines. In the case of the MDA-MB-231cell line, proliferation is not reduced as it does not have phosphorylated Her2 at tyrosine Y877. Taken together with our data, the fact that treatment with 2 µg/mL Tzm-PEG-PHis significantly reduces cell viability of MDA-MB-231, suggests another mechanism than Her2 phosphorylation.

#### 3.2.2. Migration and Invasion Assays

Due to the fact that cancer mortality is mainly related to complications of metastatic lesion, we next investigated the effect of 2 µg/mL Tzm-PEG-PHis on migration (Figure 2C) and invasion (Figure 2D). The directed movement of cells on a substrate is defined as migration [28]. Invasion of cancer cells is a more complex phenomenon and is defined as the penetration of the basement membrane and infiltration into underlying tissues, which requires adhesion, proteolysis of extracellular matrix components, and movement. For the next experimental design, we narrowed down our investigations because the free Tzm does not impair cell viability. Our data shows that in the case of the normal cell line MCF-10A, treatment with Tzm-PEG-PHis does not reduce migration or invasion (Figure 2C,D(a)) while having a strong inhibitory effect on migration and invasion on TNBC cell lines (Figure 2C,D(b,c)). Representative images are presented in Supplementary Figure S4.

### 3.3. Evaluations of Therapeutic Effects on 3D In Vitro Cell Culture

#### 3.3.1. Mammospheres Assay

**Figure 3:**
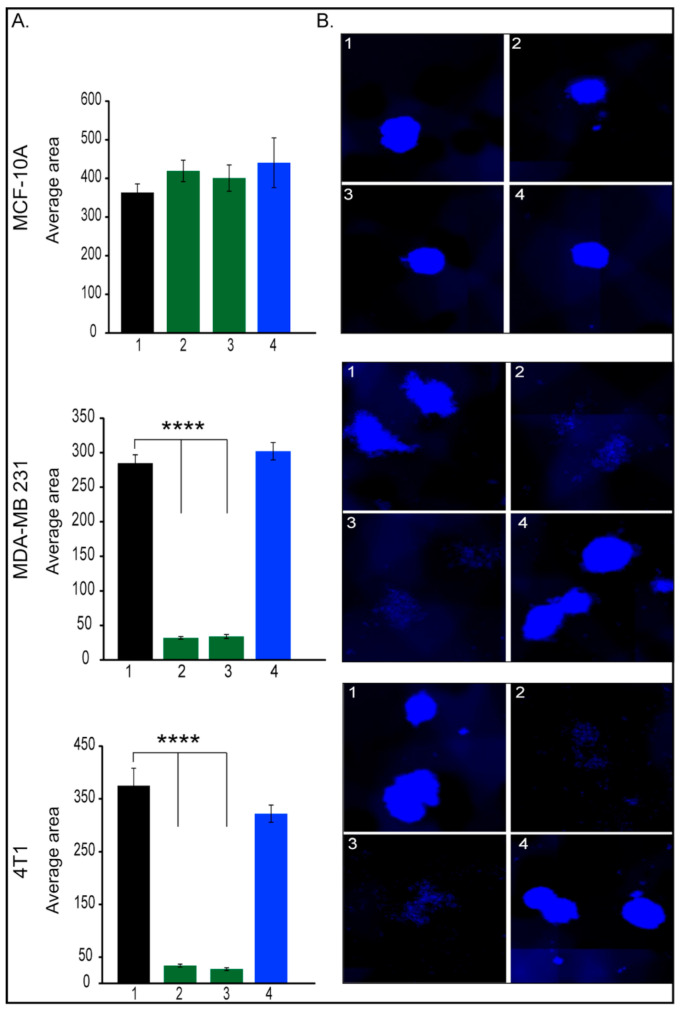
Mammosphere assay. (**A**). Quantification of mammospheres average area; (**B**). representative images of mammospheres, 1. untreated; 2. 2 µg/mL Tzm-PEG-PHis; 3. 4 µg/mL Tzm-PEG-PHis, 4. 4 µg/mL Tzm. (**B**). Representative mammospheres pictures. Pictures were acquired with 10× objective. **** *p* < 0.0001.

There is sparse evidence [29] showing that Her2 is selectively expressed in, and regulates the self-renewal of, the cancer stem cell (CSC) population in Her2-luminal breast cancers. The authors showed that among the tested cell lines, the MDA-MB-231 cell line expressed the lowest levels of Her2 and an intermediate level of aldehyde dehydrogenase (ALDH), a CSC marker. Moreover, they reported that Tzm treatment had no effect on the ALDH-positive populations in MDA-MB-231 cells. Taking into consideration the fact that breast cancer originate and is maintained by a small fraction of CSCs (also known as tumor-initiating cells) [30], which are responsible for tumor progression, metastasis, therapy resistance, and tumor recurrence, we have expanded our investigations using the mammospheres assay (Figure 3). This assay is used to propagate mammary CSCs in vitro. As we moved to the 3D investigations, we tested two concentrations of Tzm-PEG-PHis (2 µg/mL (Figure 3, columns 2) and 4 µg/mL (Figure 3, columns 3) and 4 µg/mL free Tzm (Figure 3, columns 4).

As expected, mammospheres average area has not been affected by treatments in the case of MCF-10A cell line. Moreover, in the case of tested TNBC cell lines, Tzm alone had no effect, a fact which is in line with the literature. In contrast, both concentrations of Tzm-PEG-PHis dramatically reduced mammospheres formations in both human and murine TNBC cell lines. Incubation of MDA-MB-231 with 2 or 4 µg/mL free Tzm concomitant with free micelles failed to impair mammosphere formations. Altogether, these data suggest that Tzm linked to micelles not only reduces cell viability and cell proliferation of TNBC cancer cell lines but also can impair metastatic abilities as it impairs migration, invasion, and self-renewal of CSCs. Moreover, CSCs are involved in drug resistance [31,32], and impairment of CSCs by treatment with Tzm-PEG-PHis may prevent or reduce resistance to Tzm.

#### 3.3.2. Three-Dimensional Matrigel Monocultures and Coculture

**Figure 4:**
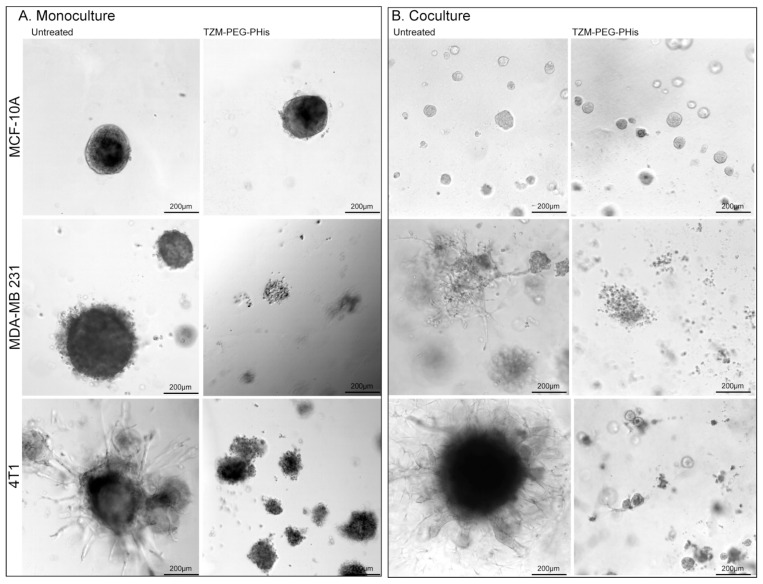
Three-dimensional Matrigel assay. (**A**). Monoculture; (**B**). coculture. MCF-10A, MDA-MB 231, and 4T1 have been cultivated together with HUVEC and fibroblast cells. Pictures are acquired using 10× microscope objective.

In 3D culture system, cells are cultivated in a Matrigel that allows them to form spheroids. Inside of the spheroids, as they grow, starvation and hypoxia occur, leading to apoptosis and the formation of microenvironmental niches. This culture system reproduces cells interaction in a more complex way. The size of the spheroids reflects cell proliferation, while invasive projections correlate with invasiveness. In our experiments (Figure 4), at 48 h after seeding the cells, we administered the first treatment followed by treatment every 7 days. A total of three weekly treatments (2 µg/mL Tzm-PEG-PHis each) were administered, and cell cultures have been fixed seven days from the last treatment for subsequent brightfield imaging.

In 3D monoculture, MCF-10A cells do not present high invasive capabilities, as the spheroids are compact with very few branches. TNBC cell lines form larger spheroids with increased invasive branches, while the murine 4T1 cell line exhibits the highest invasiveness (Figure 4A, left columns). The highest invasive ability of 4T1 relative to the MDA-MB-231 cell line observed in 3D Matrigel assay confirm the migration and invasion data presented in Figure 2, where 4T1 cell lines had a higher number of migratory and invasive cells relative to MDA-MB-231 cell lines. The 2 µg/mL Tzm-PEG-PHis did not significantly impair spheroid formations in the MCF-10A cell line, but the effect is clearly significant in TNBC cell lines (Figure 4A, right column). In treated TNBC cell lines, spheroids are smaller, less compact, and present dramatically fewer branches. However, although in 2D investigations there was similar impact on cell viability of Tzm-PEG-PHis on the human and murine TNBC cell lines, in 3D Matrigel, the treatment had a higher impact in the human TNBC cell line. This may be due to the different proliferative rates we have observed with EdU assay or by decreased ability of humanized antibody components of the Tzm-PEG-PHis micelles to interact with murine cells in 3D Matrigel model.

In living organisms, cancer cells do not grow alone; they, in fact, interact with normal cells. For example, fibroblasts promote proliferation and matrix invasion of breast cancer cells [33,34,35]. To investigate the therapeutic effects of Tzm linked to micelles, we co-seeded the tested cell lines with fibroblasts and human umbilical vein endothelial cells (HUVECs). As in monoculture, we administered three treatments at the same time intervals, but we fixed the cells one day after the last treatment, instead of seven days as we did in the monoculture investigations. The reason to do so is the fact that in the TNBC cell lines, spheroids have reached similar sizes as in monoculture. While in co-cultured MCF-10A cell line, spheroids are clearly smaller but higher in numbers, the spheroids in co-cultured TNBC cell lines are at similar sizes as with monocultured counterpart (Figure 4A,B). More importantly, the invasiveness is enhanced as the spheroids from co-cultured TNBC cell lines present higher numbers and larger invasive branches (Figure 4B, left). Treatment with 2 µg/mL Tzm-PEG-PHis significantly impaired spheroid formation, again with slightly higher impact in the case of the human TNBC cell line relative to the murine one (Figure 4B, right).

### 3.4. Evaluations of In Vivo Anti-Tumor Efficacy

#### 3.4.1. Biodistribution

We, in the in vitro experiments, used MDA-MB-231 as a representative TNBC cell line for human breast cancer and the murine equivalent of the MDA-MB-231 cell line represented by the 4T1 cell line. The murine 4T1 cell line allowed us to expand the evaluation of Tzm-PEG-PHis in vivo using Balb/c mice. The 4T1 mouse breast carcinoma cells were suspended in MEM-EBSS medium/Matrigel (1:1) (1 × 10^6^ in 50 μL) and injected into the mammary pad of female BALB/c mice under deep anesthesia, as in our previous in vivo investigations [36]. Investigating the Tzm-Her2 engagement in vitro and in vivo by using fluorescent labeled Tzm (Tzm-AF700), Rudkouskaya and co-workers [37] demonstrated that MCF10A showed no uptake of Tzm, while in MDA-MB-231 cells, Tzm–AF700 may interact with an unknown target at the cell surface of MDA-MB-231 cells, leading to its subsequent internalization. Taking into consideration the possible unspecific interaction of Tzm with other cells from living mice, for the in vivo investigations, we reduced the Tzm-PEG-PHis dose to 0.2 mg/kg b.w. per week, divided into two treatments per week. Rhodamine B (RB) from both PEG-PHis-based nanoformulations was used for biodistribution investigations.

At 2 weeks after inoculation, mice were randomly allocated into four groups: untreated tumor-bearing mice, Tzm-PEG-PHis-RB-treated group (2 µg Tzm in 0.5 mL/mouse, twice per week, i.p.), free Tzm-treated group (2 µg free Tzm in 0.5 mL/mouse, twice per week, i.p.), and the rhodamine-loaded into micelle (PEG-PHis-RB) at the corresponding concentration of micelle from Tzm-PEG-PHis-RB (0.5 mL/mouse, twice per week, i.p.). Twenty-one days after the first treatment, the mice received the last treatment, and then at 30 min after i.p. administration, they were sacrificed by neck dislocation under deep anesthesia. The primary tumors and different organs were retrieved and preserved in 10% paraformaldehyde (Sigma-Aldrich) for further analysis (histopathology and immunohistochemistry).

**Figure 5:**
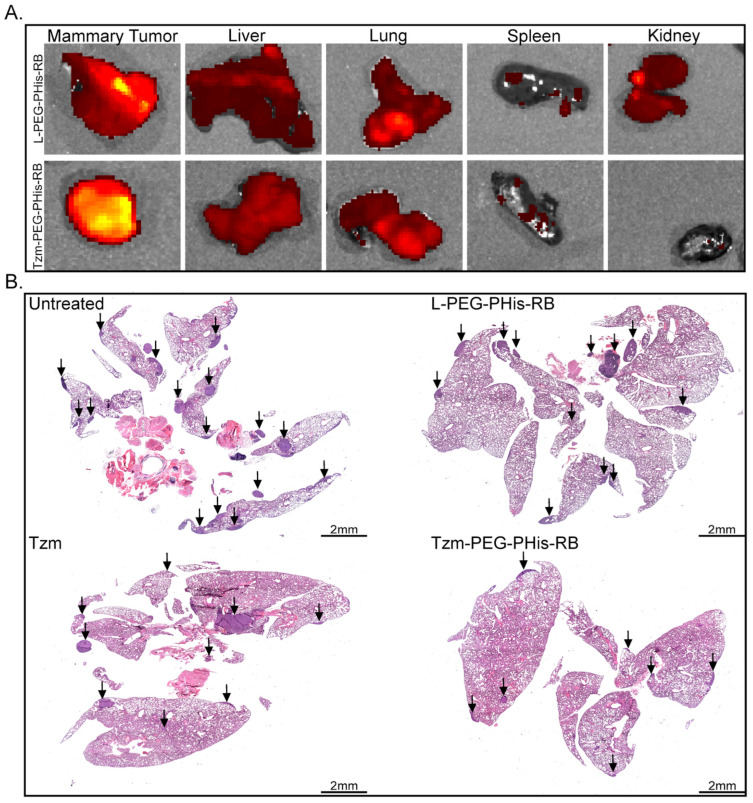
In vivo biodistribution and metastasis investigations. (**A**). Biodistribution. (**B**). HE staining of paraffin-embedded lung specimens. Arrows indicate metastases.

Fluorescent scanning of excised organs (Figure 5A) showed similar fluorescence emissions in the liver and lung, the fluorescence being very weak in the spleen, in both treated groups. In contrast, the fluorescence signal was detected in the kidney only in the case of PEG-PHis-RB, suggesting the excretion of non-targeted treatment. The fluorescence signal was stronger at the level of primary tumor of targeted-treated (Tzm-PEG-PHis-RB) mice, where RB should be unloaded and accumulated due the specific interaction of Tzm with Her2 and due to lower pH gradient inside the tumor.

Taking into consideration the fact that the lung represents the primary metastatic target of 4T1 cell line, for histological analysis, we focused on primary tumors and lungs. Hematoxylin (stain nuclei in blue) and eosin staining (stains basic components in the cytosol) revealed lung metastases (Figure 5B). Metastasis is reduced in the Tzm-PEG-PHis-RB-treated group, while treatment with free Tzm or RB-loaded micelles does not significantly impair it. The significant therapeutic effects of the Tzm-PEG-PHis-RB treatment have been further sustained by the difference in the organ’s weight and survival (Figure S5). Although we have not found a significant reduction in primary tumors (Figure S5A), the Tzm-PEG-PHis-RB treatment reduced the weight of the lungs (Figure S5B) and spleens (Figure S5C) relative to the untreated group. However, the lungs and spleens in the Tzm-PEG-PHis-RB-treated group did not reach the weight of the organs from a healthy mouse. Survival analysis of the untreated and Tzm-PEG-PHis-RB-treated groups further reflects the therapeutic effect of Tzm-PEG-PHis-RB (Figure S5D).

#### 3.4.2. Immunohistochemistry (IHC) Investigations

Among the signaling pathways involved in cancer, two pathways are frequently activated or mutated in cancer, in particular, the PI3K/AKT/mTOR signal transduction pathway and the RAS/MAPK pathway. These cascades are highly interconnected in mediating upstream signals from receptor tyrosine kinases (RTKs) to intracellular effector proteins and cell cycle regulators to promote cell growth, proliferation, differentiation, and drug resistance [38,39]. As a result, key molecules from these dysregulated pathways are therapeutic targets in cancer.

**Figure 6:**
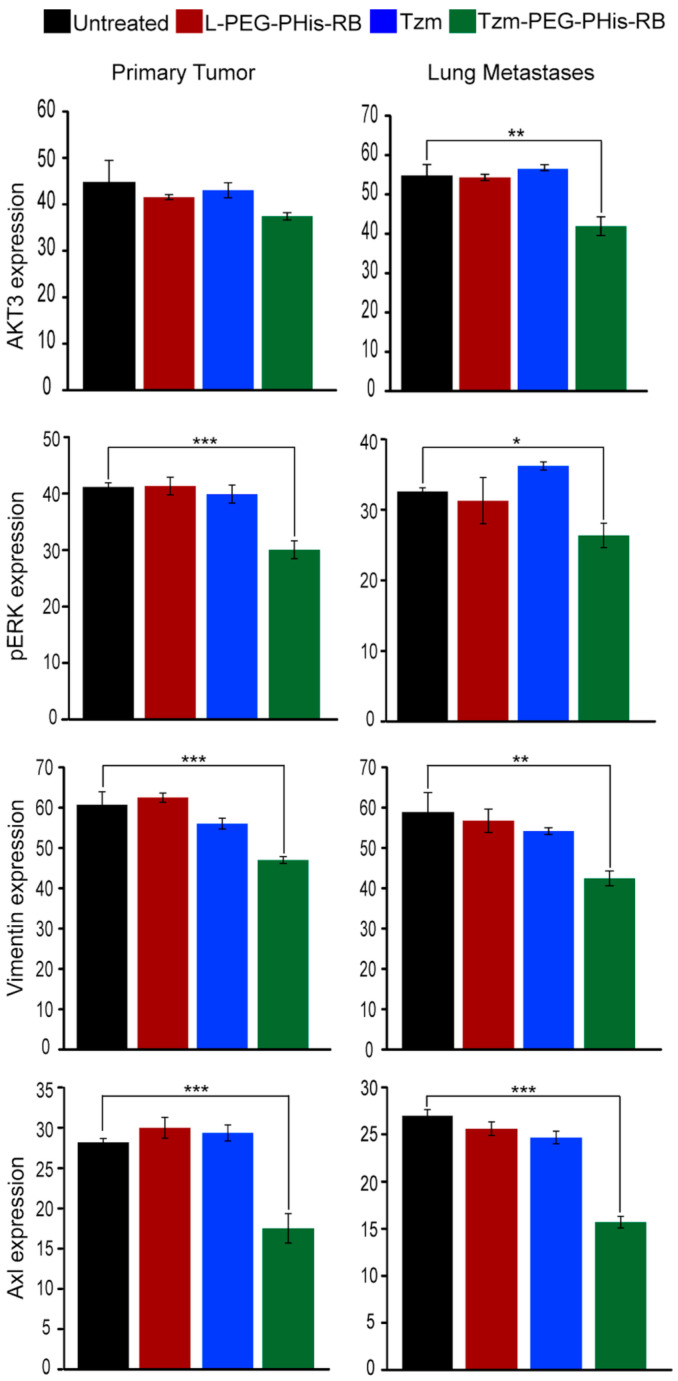
The average intensity of the IHC staining. Akt3 ** *p* = 0.0010; pErk * *p* = 0.0288, *** *p* = 0.0009; vimentin ** *p* = 0.0016, *** *p* = 0.0001; Axl *** *p* = 0.0007/0.0001. Untreated *n* = 3, L-PEG-Phis-RB *n* = 4, TZM and TZM-PEG-Phis-RB *n* = 5.

AKT, also known as protein kinase B, is a key element of the PI3K/AKT signaling pathway that mediates tumor growth, survival, and invasiveness of tumor cells. There are three Akt isoforms with distinct functions in breast cancer [40], and various inhibitors target Akt signaling in cancer [41,42]. Hinz and Jücker [40], reviewing the functions of Akt isoforms, highlight that Akt3 has a predominant effect in TNBC: ablation of Akt3 in TNBC decreases proliferation, whereas TNBC cells with Akt3 knockdown form more metastases in the lung in vivo. Moreover, although Akt3 is required for TNBC proliferation and tumor growth, it does not promote an invasive phenotype [43]. Having this in mind, we have performed IHC staining for Akt3 (Figure 6). Our data did not identify major changes in the expression of Akt3, neither in primary tumors nor in lung metastases, in the free Tzm or PEG-PHis-RB-treated groups relative to untreated mice. However, in lung metastases, Akt3 expression is significantly reduced in the Tzm-PEG-PHis-RB group. This data is supported by the literature and endorses our data related to lung metastases as shown by HE staining. Representative images of Akt3 IHC staining for primary tumors and lung sections are presented in Supplementary Figure S6A. The IHC Akt3 data from the primary tumors explains the lack of size reduction observed by weighing the tumors or by HE sections size.

The second main signaling pathway, the RAS/MEK/ERK pathway, regulates diverse cellular processes such as cell proliferation, differentiation, motility, and survival. Activation of ERK1/2 generally promotes cell proliferation, and its deregulated activity is a hallmark of many cancers. In TNBC, phosphorylation of ERK induces epithelial to mesenchymal transition (EMT) required for metastases by increasing expression of Slug, vimentin (EMT marker), and Axl receptor and decreasing expression of E-cadherin [44]. Genetic and pharmacological evidence suggests that ERK signaling mostly provides pro-survival cues in response to serum and RTK activation. However, ERK1/2 activation can also inhibit apoptosis induced by the death receptors Fas or TNF (extrinsic death pathway) [45]. Using IHC staining, we found that only the Tzm-PEG-PHis-RB treatment presented biological effects on the level of the phosphorylated ERK (pERK1/2) (Figure 6). In contrast to expression pattern of Akt3, the reduction in pERK1/2 was observed at the level of both primary tumors and lung metastases. The pERK1/2 data endorses migration, invasion, and cell viability/proliferation investigations assessed by 2D cell culture. Representative images of pERK1/2 IHC staining for primary tumors and lungs sections are presented in Supplementary Figure S6 (Figure S6B).

Our in vitro data demonstrated the impairment of invasiveness of tested TNBC cell line by treatment with 2 µg/mL Tzm-PEG-PHis. Invasion involves EMT that allows a polarized epithelial cell, in contact with the basement membrane, to switch to a mesenchymal phenotype and migrate away from the original epithelial layer. The markers used to highlight EMT are increased vimentin and decreased E-cadherin expression. Vimentin, a cytoskeleton protein associated with a migration phenotype, is required for TNBC metastasis and represents a poor prognostic factor for TNBC [46,47]. In order to validate the in vitro invasiveness data, in the subsequent investigations, we have stained paraffin-embedded sections using IHC for vimentin expression (Figure 6). The expression of vimentin followed the expression pattern of pERK1/2, strengthening the data regarding the impairments of metastasis via pERK1/2–vimentin cascade. The vimentin expression was significantly decreased only in the Tzm-PEG-PHis-RB-treated group. Representative images of vimentin IHC staining for primary tumors and lungs sections are presented in Supplementary Figure S7 (Figure S7A).

In TNBC, as cells lack ER and PR, other receptors become responsible for mediating upstream signals. Axl receptor has been found to be significantly upregulated in TNBC cell lines [48], to be a mediator of cellular growth and survival [49], to be an essential EMT-induced regulator of breast cancer metastasis and patient survival [50], and to control directed migration of mesenchymal TNBC cells [51]. Taking into consideration the above-mentioned data, we have used IHC to stain for Axl expression (Figure 6). In line with the pERK1/2 data and the reviewed data by Wu and co-authors [44], treatment with Tzm-PEG-PHis-RB significantly reduced the expression of Axl in both primary tumors and in lung metastases. Representative images of Axl IHC staining for primary tumors and lungs sections are presented in Supplementary Figure S7 (Figure S7B).

#### 3.4.3. Ex Vivo Cell Death Assessment by TUNEL

**Figure 7:**
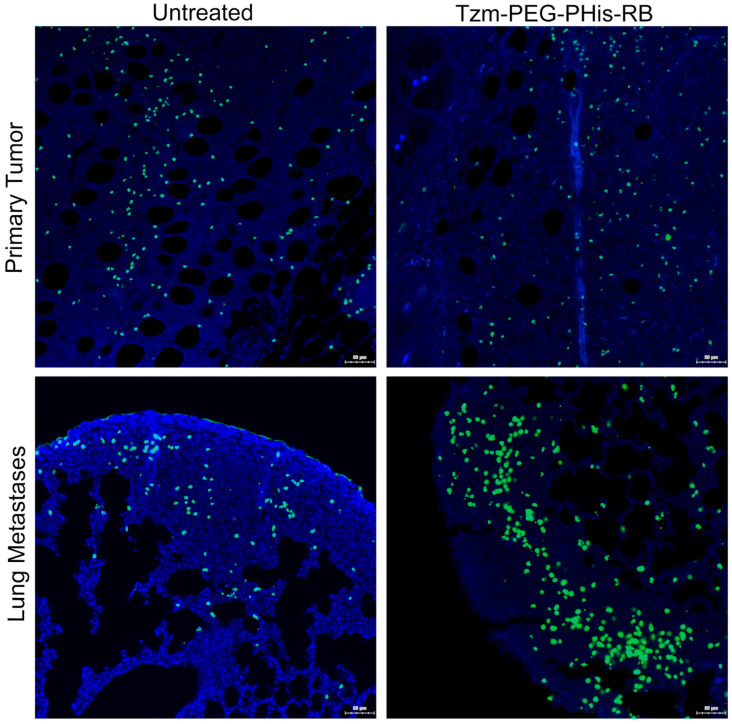
TUNEL staining. Blue—nuclei; green—death cells.

Cancer development is a result of both cell proliferation and cell death. The impairment of Akt3 (in lung metastases) and pERK1/2 (both in primary tumors and lung metastases) should reflect the biological processes that they modulate. In the next experimental design, we have deployed TUNEL assay in order to investigate ex vivo cell apoptosis (Figure 7).

Fluorescent signal of stained dead cells shows no significant difference between untreated and Tzm-PEG-PHis-RB-treated primary tumors at the tumor invasion front. This data, together with the fact that primary tumors are relatively the same size across the groups, support the Akt3 IHC data, as the tumor proliferation and anti-apoptotic Akt3 expression are not decreased in treated groups relative to the untreated group. In contrast, at the level of lung metastases, Akt3 expression is decreased and provides the molecular support of the TUNEL data, which shows significantly increasing number of dead cells in lung metastases treated with Tzm-PEG-PHis-RB relative to the untreated mice. TUNEL assay reveals the dead cell. However, we lack data regarding the percentage of dying cells.

There is data showing that it is possible for the Her2 status to change between primary tumors and paired metastases, shifting from Her2-negative primary tumors to Her2+ [52] or to Her2 1+ or 2+ metastases [53]. Moreover, Her2-positive circulating tumor cells have been detected in patients with Her2-negative primary tumors [54]. At this moment, we are not aware if this phenomenon is responsible for the higher impact of Tzm-PEG-PHis-RB at the level of lung metastases vs. primary tumors, or the tested concentration was too low to have a significant impact on primary tumor development. In addition, we cannot exclude the lower efficiency of Tzm therapeutic effects in mice, as the Tzm has been designed for human use.

### 3.5. Western Blot Investigations

**Figure 8:**
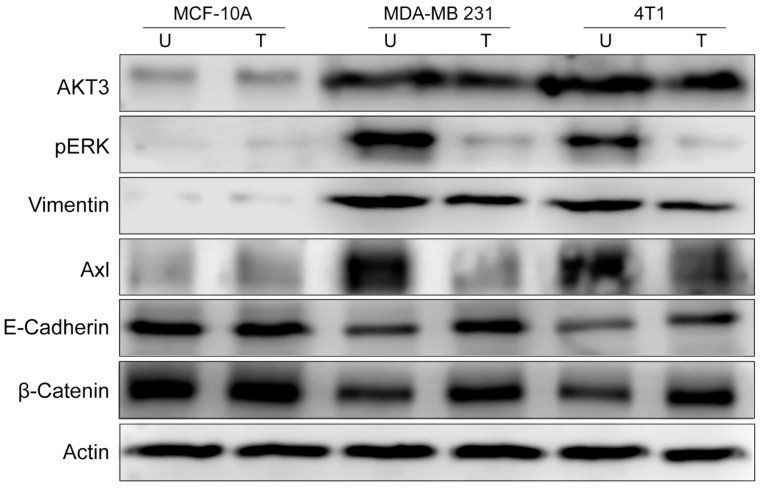
Western Blot investigations. U—untreated; T—treated with 2 µg/mL Tzm-PEG-PHis. Actin is used as a loading control.

Next, we aim to confirm by Westen Blot (WB) the findings from in vivo experiments. Using cell homogenates obtained from 2D cell cultures, we found by WB a low expression of Akt3, pERK1/2, vimentin, and Axl in MCF-10A cell line (Figure 8) and elevated levels in TNBC untreated cell lines. Treatment with 2 µg/mL Tzm-PEG-PHis for 72 h impaired their levels only in TNBC cell lines, confirming the IHC data from the ex vivo experimental design.

As the invasiveness is decreased by Tzm-PEG-PHis treatments, we wonder if this involves only impairment of EMT or if it reverses it by inducing the mesenchymal to epithelial transition (MET). EMT is not always full EMT; in partial EMT, cells co-express epithelial and mesenchymal markers or lose epithelial markers without gaining mesenchymal markers [55]. Western Blot investigations of E-cadherin (an epithelial marker) revealed, as expected, high levels in the epithelial MCF-10A cell line and lower levels in untreated mesenchymal TNBC cell lines. Treatment with 2 µg/mL Tzm-PEG-PHis increased E-cadherin levels in TNBC cell lines, the effect being stronger in human MDA-MB-231 cell line. Alone, only vimentin and E-Cadherin expression patterns do not reveal if it is a partial EMT or MET. Using transformed breast epithelial cells, Li and Mattingly [56] demonstrated that inhibition of MAPK, but not PI3-kinase, was able to completely restore E-cadherin cell–cell junctions and epithelial morphology in cell lines with moderate H-Ras expression. In MCF10A cells transformed by high-level expression of activated H-Ras or N-Ras, restoration of E-cadherin junction required both the enforced re-expression of E-cadherin and suppression of MAPK kinase. Enforced expression of E-cadherin alone did not induce reversion from the mesenchymal phenotype. Moreover, inhibition of ERK /MAPK activation causes a modest increase in the expression of β-catenin because it can stabilize functional E-cadherin complexes at the cell surface [56]. Following this clue, we found by WB that in TNBC cell lines 2 µg/mL Tzm-PEG-PHis treatment resulted in increasing protein levels of β-catenin.

Our data, decreasing vimentin expression concomitant with increasing E-Cadherin and β-catenin and impairment of pERK1/2 expression, suggest that treatment with Tzm-PEG-PHis induces MET on TNBC cell line rather than partial EMT.

**Figure 9:**
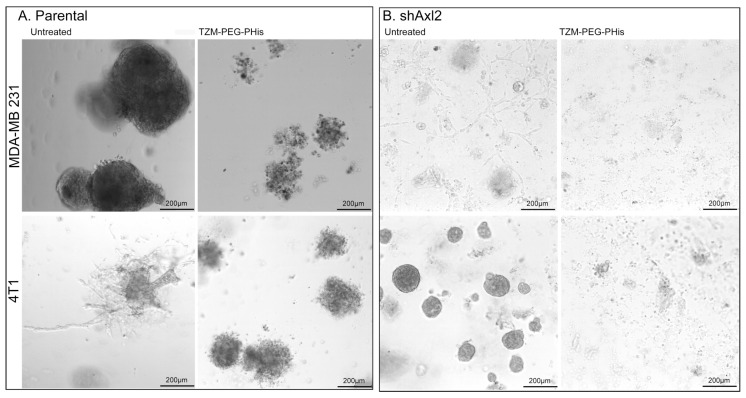
Three-dimensional Matrigel assay. (**A**). parental TNBC; (**B**). shAxl2 TNBC.

Her2 is an orphan receptor without a ligand. Through ligand-induced heterodimerization, all Her receptors could be fully activated to mediate cell signaling; Her2 spontaneously forms homodimers when overexpressed in cells, and all the other Her receptors dimerize preferably with Her2, as reviewed by Nami and co-authors [57]. EGFR/Her2 heterodimers play a key role in modulation of Tzm effects in Her2+ cell lines SK-BR3 and BT474 [58], while Her2/Axl heterodimerization is responsible for trastuzumab resistance in Her2+ breast cancer cell lines [59]. In Her2 negative cells, due to the non-overexpression of Her2 but overexpression of Axl or EGFR, there is a higher probability to find Her2 heterodimers rather than homodimers; overexpressed Axl and EGFR may be the preferred partners for Her2 heterodimers. In order to investigate the role of Her2/Axl heterodimerization in mediating the effects of Tzm-PEG-PHis, we have assessed the effects of single treatment of 4 µg/mL Tzm-PEG-PHis in both TNBC cell lines tested and on MDA-MB-231 and the 4T1 cells lacking Axl overexpression (shAxl2) (Figure 9).

While the results on parental TNBC cell lines (Figure 9A) resemble those of previous monoculture assays, with some differences due to experimental design (concentrations, number of treatments, and length of experiment), in the case of shAxl2, we observe a decrease in the size and of the projections of the spheroids (Figure 9B). In the presence of single 4 µg/mL Tzm-PEG-PHis treatment, two weeks after treatment administering the shAxl2, spheroids were insignificant, and on the Matrigel, there were cellular debris. Tzm-PEG-PHis binds to Her2 receptor, and the presence of micelles may prevent the interaction of adjacent Axl or EGFR receptors to interact with their ligands, blocking both signaling pathways. Tzm linked to micelles specifically target the Her2 receptor, but the overall effect is due to the unspecific prevention of binding the ligands of heterodimer partners or other neighborhood receptors by the presence of micelles.

## 4. Discussion

Trastuzumab (Herceptin) is currently used as an adjuvant treatment of metastatic Her2-overexpressing breast cancer. Patients with Her2- negative breast cancer do not benefit from Tzm therapy. Recently, the Food and Drug Administration (FDA) approved Tzm deruxtecan (Enhertu) for the treatment of Her2-low breast cancers that cannot be removed surgically or that have metastasized. Our data demonstrates that Tzm linked to PEG-PHis micelles is highly efficient as a therapeutic agent in the case of our TNBC pre-clinical cancer models, impairing metastasis even at lower doses than clinically used for Her2+ patients. Our findings may allow the extension of Tzm therapy to other patients, Her2-negative, that currently do not benefit from Tzm therapy. Even more, the impact of Tzm linked to micelles on CSCs may prevent the induction of treatment resistance. In the current manuscript, the micelles have been loaded only with rhodamine; however, loading various biological active molecules can further modulate the therapeutic effect of Tzm-PEG-PHis nanostructures. These molecules may target Her2 heterodimers or key signaling pathways, depending on the specificity of cancer cell characteristics. Tzm linked micelles functionalized by loading doxorubicin have also been tested both in vitro and in vivo, in Her2+ cells [60]. Using JIMT-1 tumor cultures (which express moderate levels of HER2, but it is hardly sensitive to Tzm treatment), the authors tested empty micelles with or without Tzm at an equivalent concentration of 4 μg/mL of Tzm. Both variants reduced the growth of explants, but the Tzm-conjugated variant induced higher toxicity than the unconjugated variant. Moreover, only the TZM-conjugated micelles displayed a reduced cell proliferation in JIMT-1 cells. In the case of HBCx39 PDX cultures (HBCx39 tumors possess a relatively low expression of HER2 and is used because it is responsive to other Tzm-based therapeutics), Tzm-conjugated DOX-loaded micelles did not show any improved effects compared to the unconjugated counterpart or free DOX. Altogether, the data shows that the effects of Tzm-linked micelles depend on the cell type, even when they are unresponsive to treatment with Tzm alone. The receptor expressed on the cells surface vary, one reason is represented by the cell type. Tested TNBC compensate the signaling arrived trough ER, PR and overexpressed Her2 receptors by using other type of receptors, Axl being one that is overexpressed in MDA-MB 231 TNBC cell line. We believe that Tzm-Linked to micelles, due to the presence of the micelles prevent the agonist to interact to various receptors of the cancer cell, the mechanism being unspecific. As the following Her2+ cell lines express same ER, PR and Her2 status but have opposite reactions to the trastuzumab treatment, SK-BR3 (ER-, PR-, Her2+, responsive to trastuzumab) and HCC 1954 (ER-, PR-, Her2+, non-responsive to trastuzumab), we expect that various TNBC cell lines to have various interactions with Tzm-linked micelles depending on their receptors. This means that the micelles must be loaded with various active biomolecules, depending on the particularities of the cancer cells to further modulate these interactions.

## 5. Conclusions

Our in vitro and in vivo data demonstrates that Tzm linked to PEG-PHis micelles possesses anti-tumoral effects in the investigated TNBC cell lines. However, using only one type of representative cell line for normal, human triple-negative, and murine triple-negative cell lines, along with a single animal model with reduced animal numbers for each group (to comply to with the 3R rules), prevents us from concluding if the indicated mechanism occurs in various clinical situations. Additional significant investigations (e.g., more types of TNBC cell lines, modulation of the immune system or patient-derived xenografts (PDX) models) must be deployed in order to fully demonstrate the mechanism of action. Although our data demonstrate the anti-tumoral effects in the selected TNBC models, there is a need of more data in order to investigate the effects of functionalized nanostructures into the patients, were is an even higher variety of TNBC types.

## Data Availability

All the presenting data is available within the article or Supplementary Files.

## Supplementary Materials

The following supporting information can be downloaded at: https://www.mdpi.com/article/10.3390/pharmaceutics17121554/s1, Figure S1: STEM images of PEG PHis micelles (a), Linker PEG PHis (b), Tzm PEG PHis (c) with enlargement of the area in the image (c) where the attachment of Tzm to the surface of the micelles is highlighted (d); Figure S2: Cell viability of MCF 10A (A) and MDA MB 231 (B) cell lines. Ctr untreated, PEG PHis (empty micelles), Tzm PEG PHis (Tzm linked to micelles), Tzm (free Tzm). **** *p* < 0.0001; Figure S3: Cell proliferation via EdU staining. A. Representative pictures, B. EdU positive cells expressed as % of total cells. 1. MCF 10A untreated, 2. MCF 10A treated with Tzm PE PHis, 3. MDA MB 231 treated with Tzm PE PHis, 4. 4T1 treated with Tzm PE Phis; Figure S4: Representative pictures of migration (A) and invasion (B) assays. Pictures of stained nuclei are acquired using 10× microscope objective. Quantifications of the nuclei are ploted in Figure 1C,D; Figure S5: Excised organs weight (grams) (A–C) and survival (D). A. Primary tumors, B. Lungs, C. Spleens, D. Comparative survival of untreated vs Tzm PEG PHis RB treated mice. * *p* = 0.0212, ** *p* = 0.0062; Figure S6: Representative pictures of IHC stained paraffin embeded primary tumors and lung specimens. A. Akt3 protein expression, B. pERK1/2 protein expression; Figure S7: Representative pictures of IHC stained paraffin embeded primary tumors and lung specimens. A. Vimentin protein expression, B. Axl receptor expression.

## Abbreviations

The following abbreviations are used in this manuscript:

Tzm	Trastuzumab
EMT	epithelial to mesenchymal transition
MET	mesenchymal-to-epithelial transition
TNBC	triple-negative breast cancer
ER	estrogen receptor
PR	progesterone receptor
Her2	human epidermal growth factor receptor 2
PEG	poly ethylene glycol
PHis	poly histidine
STEM	Scanning transmission electron microscopy
EDTE	Ethylenediaminetetraacetic acid
BSA	bovine serum albumine
EDU	5-ethynyl-2′-deoxyuridine
shAxl2	Axl knockdown
BB	blocking buffer
TBST	Tris-buffered saline Tween
ALDH	aldehyde dehydrogenase
CSC	cancer stem cell
HUVEC	human umbilical vein endothelial cells
RB	rhodamine B
PI3K	Phosphoinositide 3-kinase
AKT	Protein kinase B
mTOR	mammalian target of rapamicyne
IHC	immunohistochemistry
IF	immunofluorescence
RAS	Rat Sarcomas
MEK	mitogen-activated protein kinases
ERK	extracellular signal-regulated kinases
pERK1/2	phosphorylated ERK
TUNEL	Terminal deoxynucleotidyl transferase dUTP nick end labeling
EGFR	epidermal growth factor receptor
DAPI	diamidino-phenylindole
