# Supplementary material for: Trastuzumab-Conjugated pH-Sensitive Micelles Exhibit Antitumor Activity and Induce Mesenchymal-to-Epithelial Transition in Triple-Negative Breast Cancer Cell Lines
Source: Pharmaceutics. 2025 Dec 2;17(12):1554. doi: 10.3390/pharmaceutics17121554 (PMC12736354; doi:10.3390/pharmaceutics17121554)
Supplement: Supplementary file 1 [file pharmaceutics-17-01554-s001.zip › pharmaceutics-3942123-supplementary.pdf]

Supporting information

Trastuzumab conjugated pH-sensitive micelles exhibit antitumor activity and induce mesenchymal to epithelial transition in triple-negative breast cancer cell lines

Crina Elena Tiron[1], Gabriel Luta[1], Razvan Ghiarasim[2], Adrian Tiron[1,], Valentin Nastasa[3], Dragos Constantin Anita[3], Tore Geir Iversen[4,5], Tore Skotland[4,5], Kirsten Sandvig[4,5,6], Mihai Mares[3], and Mihail-Gabriel Dimofte[1,7]*

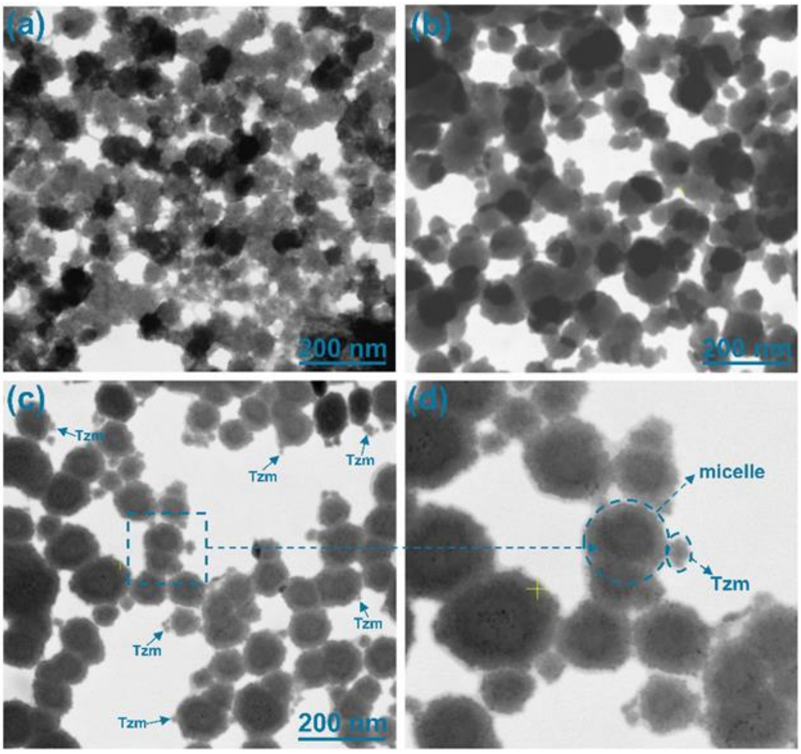

Figure S1. STEM images of PEG-PHis micelles (a), Linker-PEG-PHis (b), Tzm-PEG-PHis (c) with enlargement of the area in the image (c) where the attachment of Tzm to the surface of the micelles is highlighted (d).

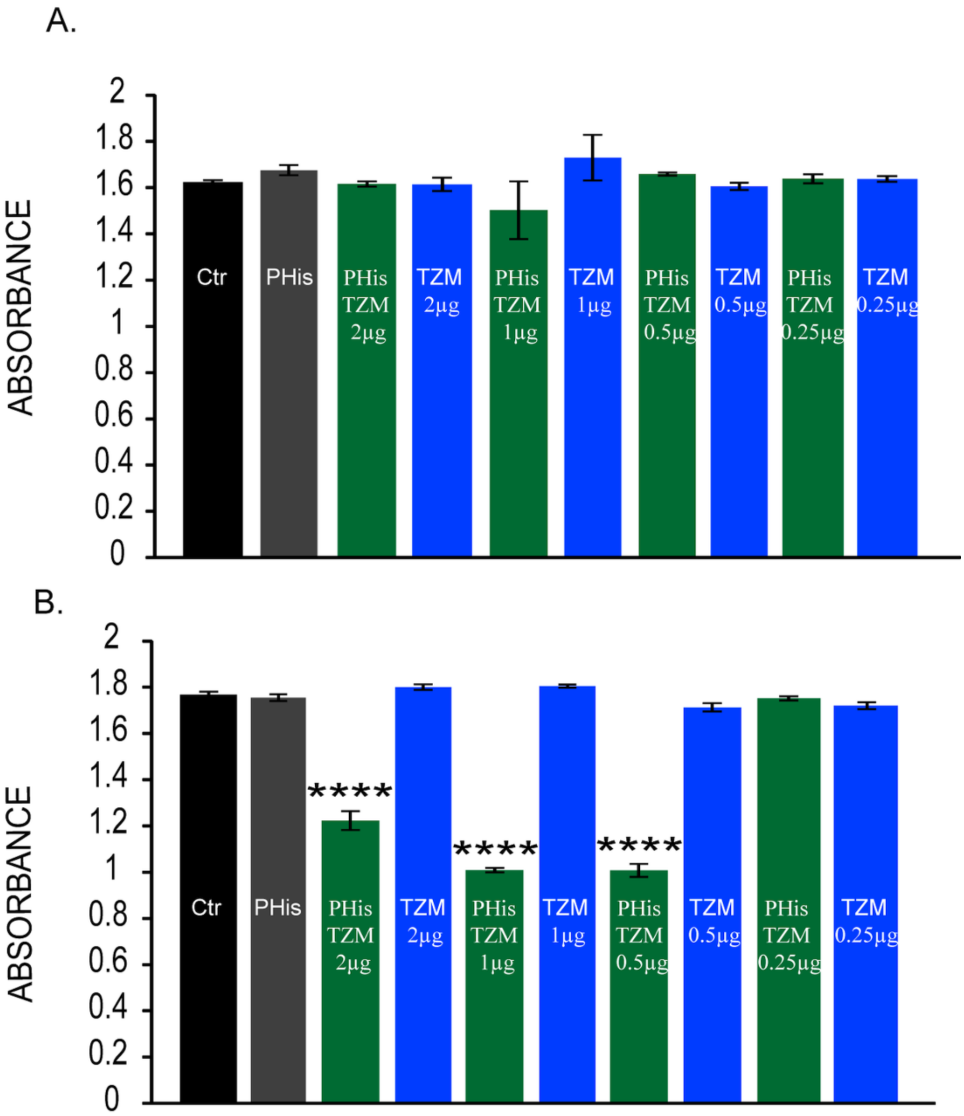

Figure S2. Cell viability of MCF-10A (A) and MDA-MB 231 (B) cell lines. Ctr – untreated, PEG-PHis (empty micelles), Tzm-PEG-PHis (Tzm linked to micelles), Tzm (free Tzm). **** p< 0,0001

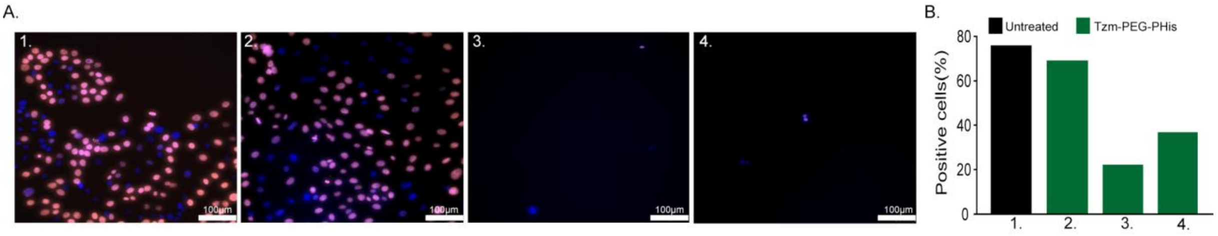

Figure S3. Cell proliferation via EdU staining. A. Representative pictures, **B.** EdU positive cells expressed as % of total cells. **1**. MCF-10A untreated, **2.** MCF-10A treated with Tzm-PE-PHis, **3.** MDA-MB 231 treated with Tzm-PE-PHis, **4.** 4T1 treated with Tzm-PE-PHis.

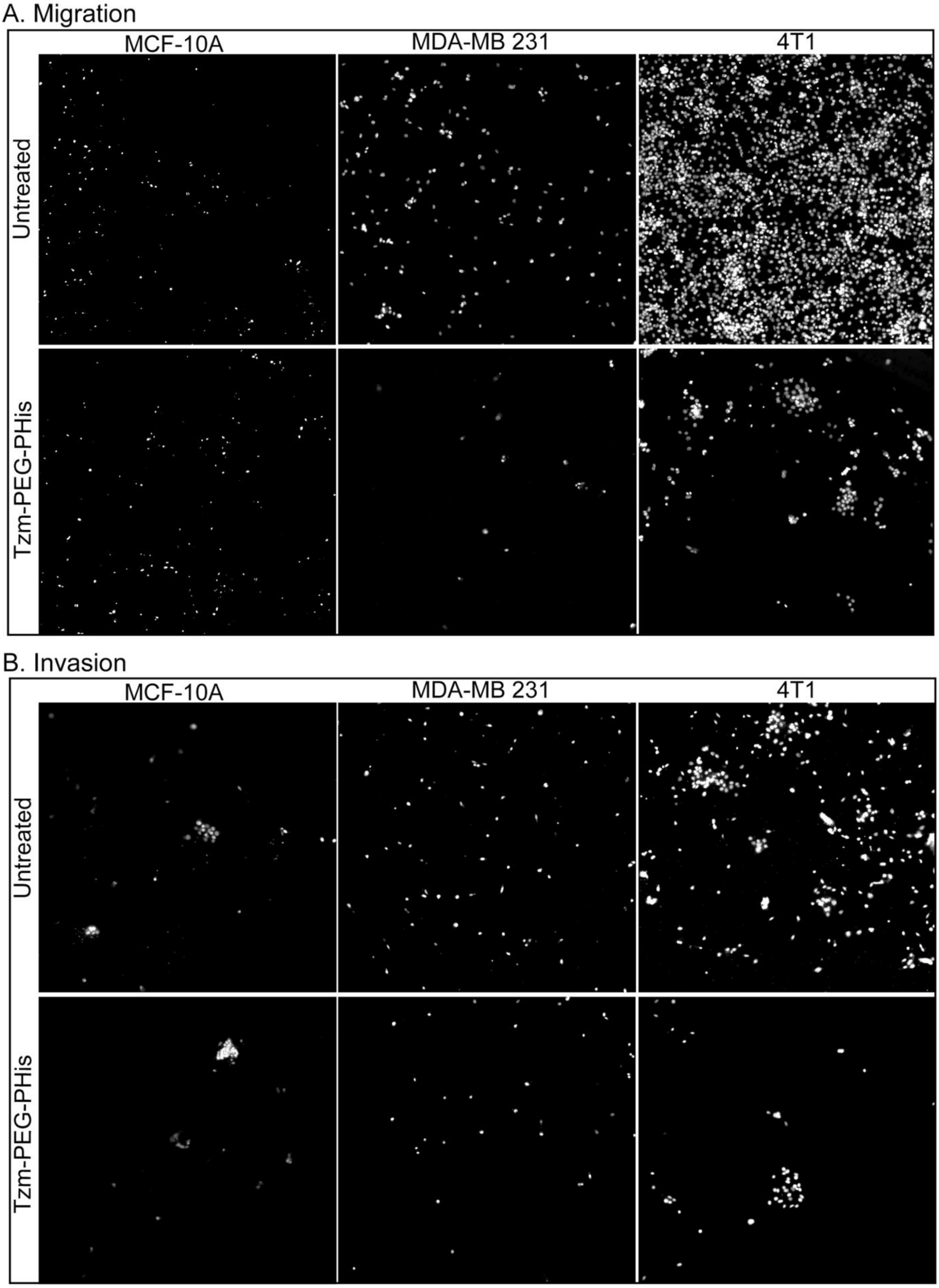

Figure S4. Representative pictures of migration (A) and invasion (B) assays. Pictures of stained nuclei are acquired using 10x microscope objective. Quantifications of the nuclei are ploted in Fig. 1 C and D.

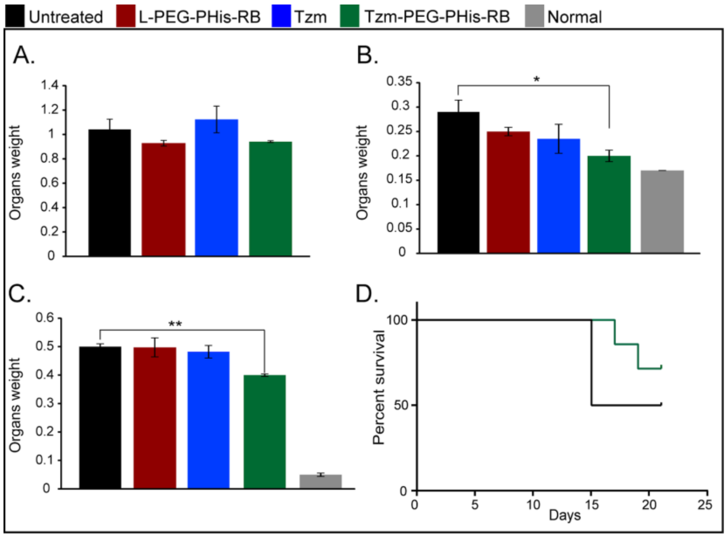

Figure S5. Excised organs weight (grams) (A-C) and survival (D). A. Primary tumors**, B.** Lungs, **C.** Spleens, **D.** Comparative survival of untreated vs Tzm-PEG-PHis-RB treated mice. * p = 0,0212, ** p = 0,0062.

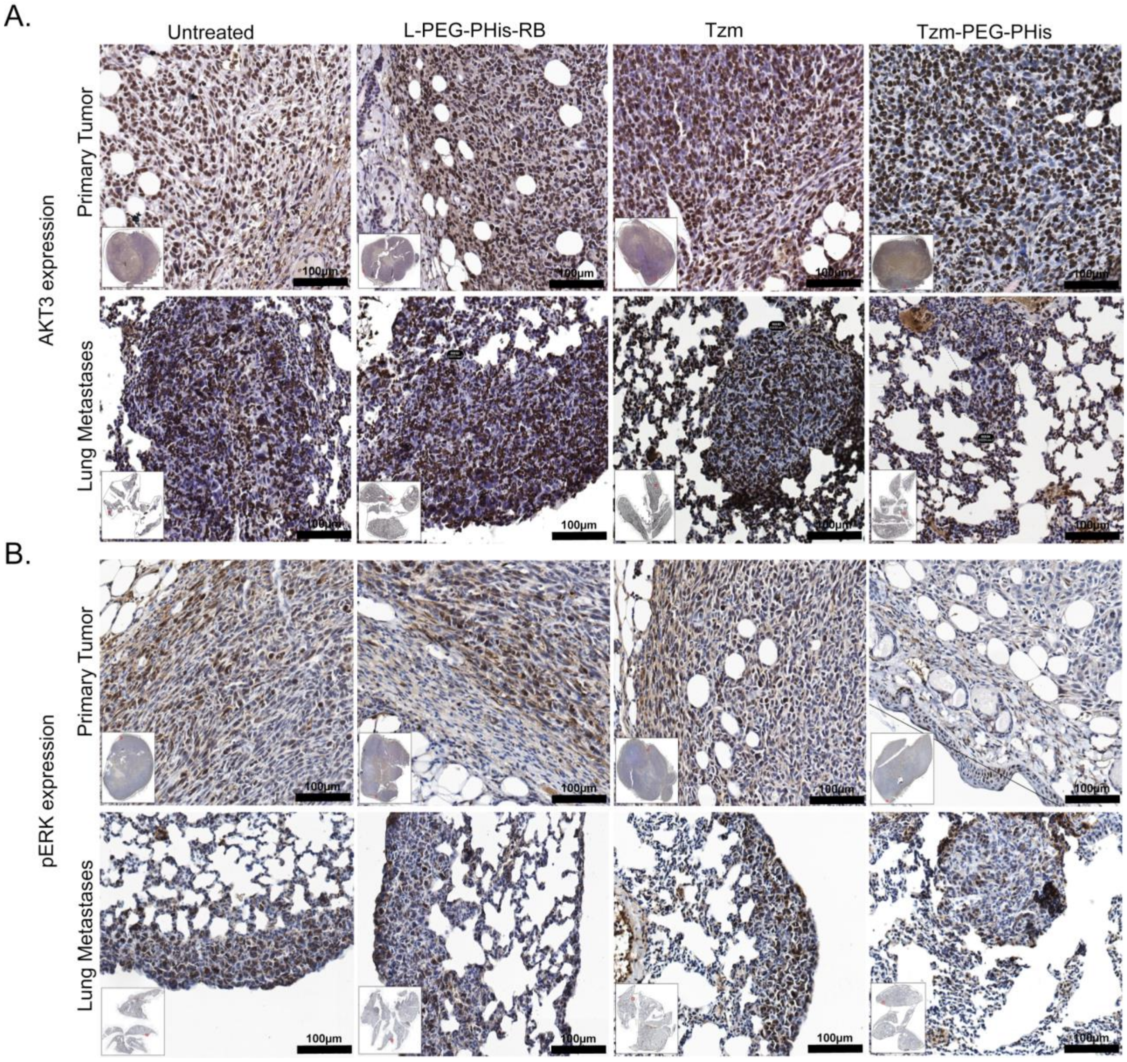

Figure S6. Representative pictures of IHC stained paraffin embeded primary tumors and lung specimens. A. Akt3 protein expression, **B.** pERK1/2 protein expression.

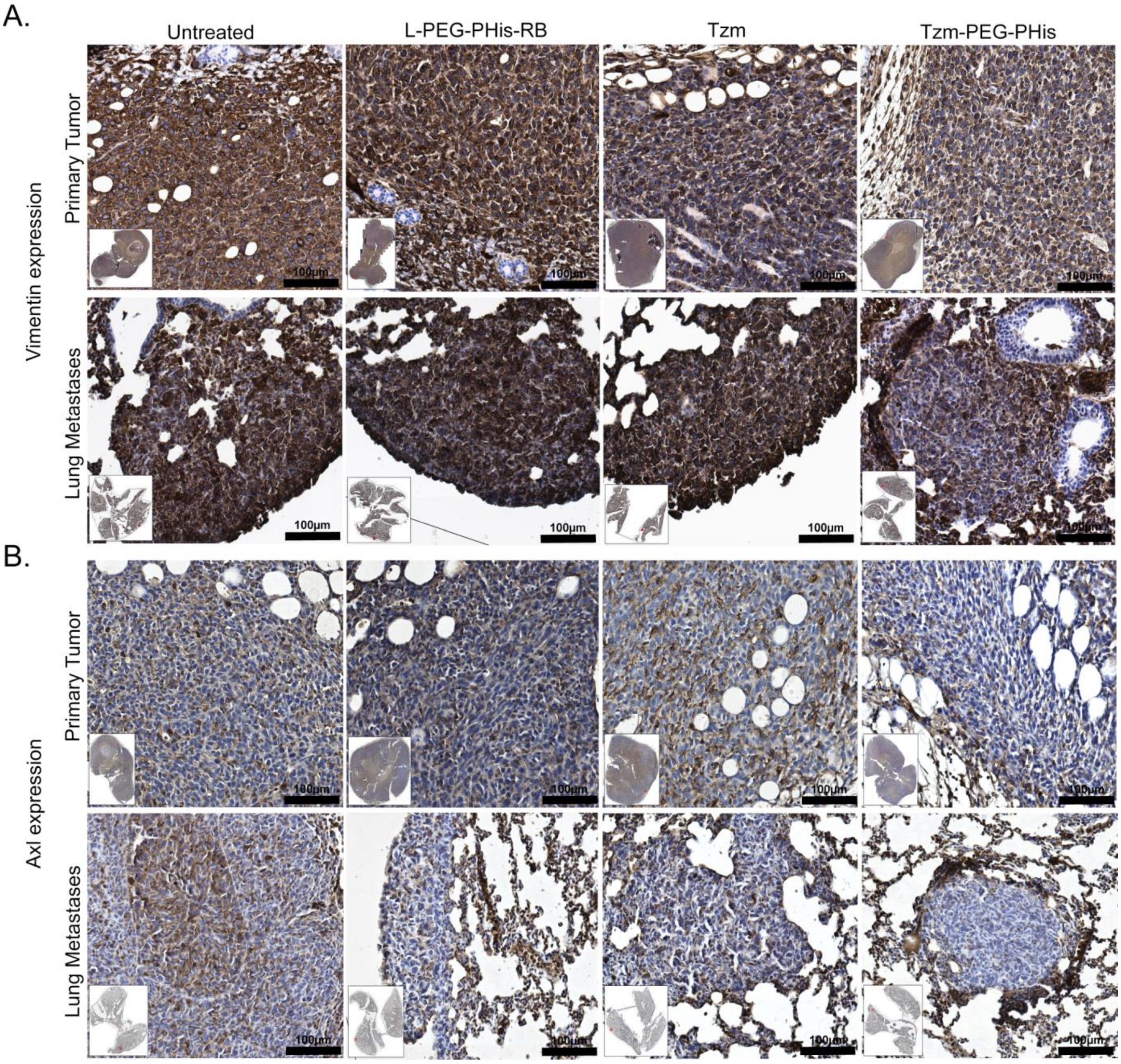

Figure S7. Representative pictures of IHC stained paraffin embeded primary tumors and lung specimens. A. Vimentin protein expression, **B.** Axl receptor expression.

**Akt3 blot**	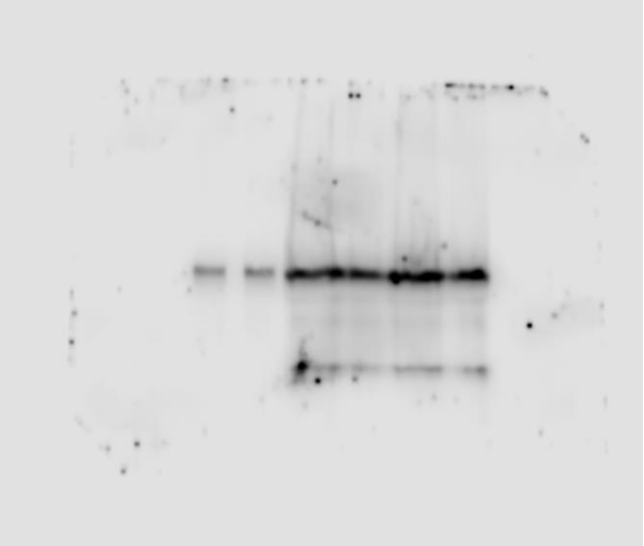

**pERK blot**	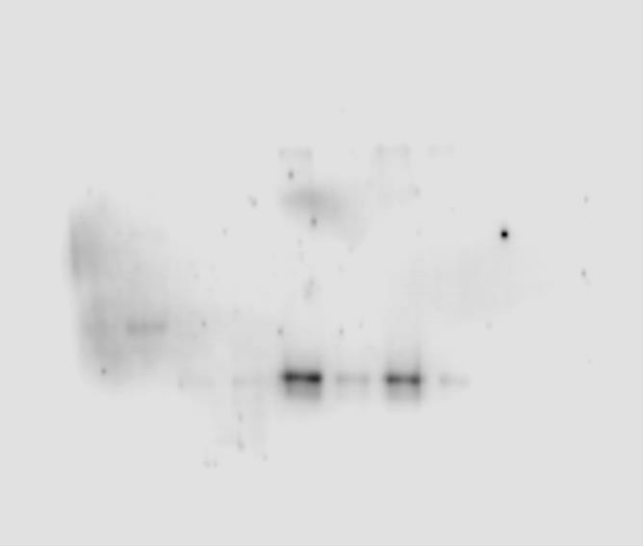
**Vimentin blot**	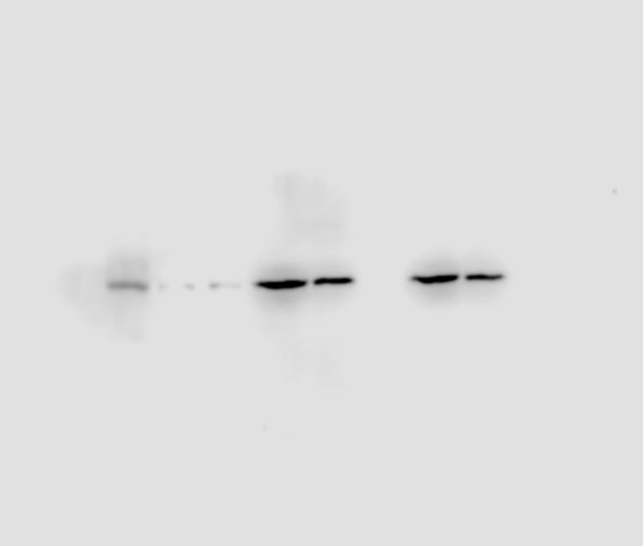
**Axl blot**	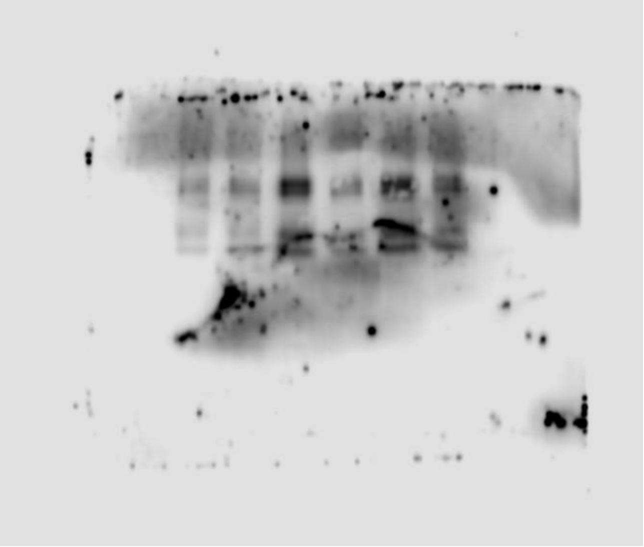

**E-Cadherin blot**	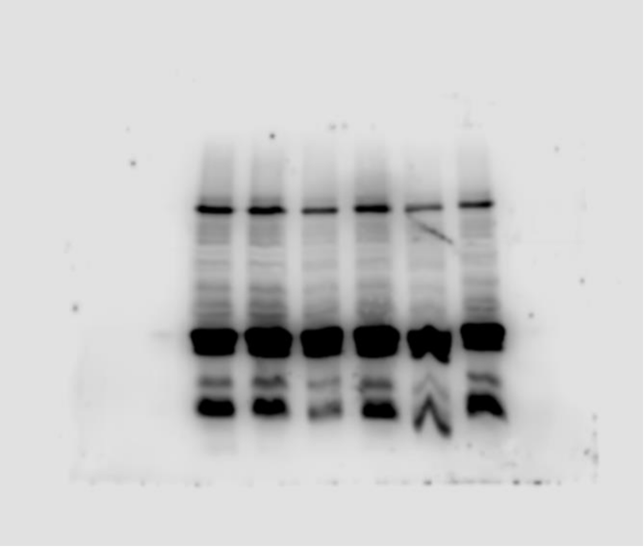
**β-Catenin blot**	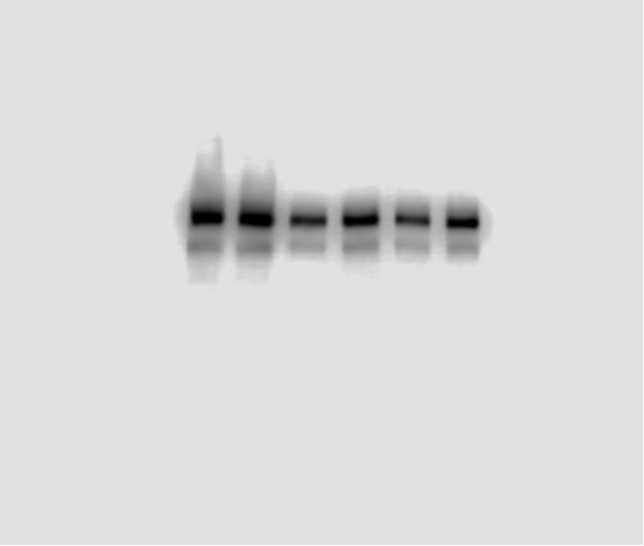
**Actin blot**	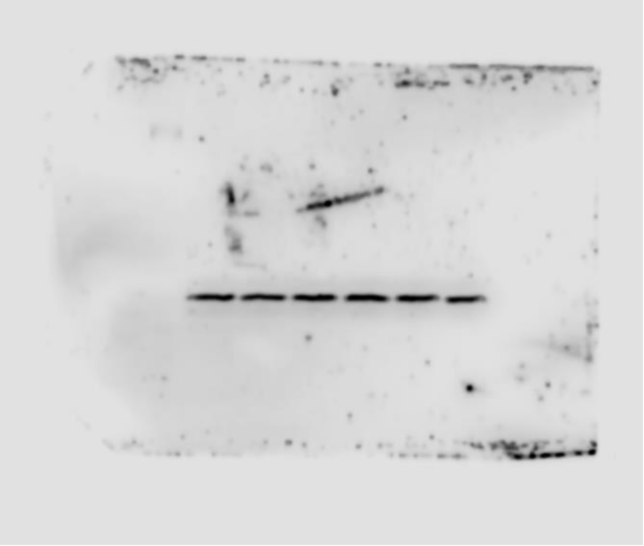
